# Puzzle of highly pathogenic human coronaviruses (2019-nCoV)

**DOI:** 10.1007/s13238-020-00693-y

**Published:** 2020-02-22

**Authors:** Jing Li, Wenjun Liu

**Affiliations:** CAS Key Laboratory of Pathogenic Microbiology and Immunology, Institute of Microbiology, Chinese Academy of Sciences, 100101, Beijing, China; University of Chinese Academy of Sciences, 100049, Beijing, China; CAS Key Laboratory of Pathogenic Microbiology and Immunology, Institute of Microbiology, Chinese Academy of Sciences, 100101, Beijing, China; University of Chinese Academy of Sciences, 100049, Beijing, China; Institute of Microbiology, Center for Biosafety Mega-Science, Chinese Academy of Sciences, 100101, Beijing, China

2019-nCoV is identified as the cause of an outbreak of viral pneumonia first detected in Wuhan, China and it continues to spread. We summarized the characteristics of genes and receptors, antiviral therapy, and early warning and prediction. We put forward the importance of vaccine development and recommend that relevant research be carried out in specific laboratories.

A novel coronavirus, designated 2019-nCoV, by the World Health Organization, is identified as the cause for an outbreak of viral pneumonia first detected in Wuhan city of China and it continues to spread throughout the world (Perlman, 2020; Zhu et al., 2020). These viruses have a characteristic “crown” structures observed by electron microscopy and are classified under the family *Coronaviridae* within the order *Nidovirales*. The coronavirus genome is composed of a positive-sense, single-stranded RNA of about 30Kb, which is the largest non-segmented genome among known RNA viruses. It can cause respiratory, digestive, and nervous system diseases in humans and animals (Hilgenfeld and Peiris, 2013). Initially, few cases of viral pneumonia were reported in Wuhan, the capital of Hubei province, in December 2019, and most of the patients affected had visited a common local seafood and animal market, which suggested animal-to-person transmission (Huang et al., 2020; Wang et al., 2020). However, it was later reported that a growing number of patients did not have exposure to animal markets, indicating that a person-to-person spread is occurring (Chan et al., 2020). This outbreak brought back memories of the severe acute respiratory syndrome coronavirus (SARS-CoV) outbreak in China in 2003, and the Middle East Respiratory Syndrome coronavirus (MERS-CoV) in Saudi Arabia in 2012 (Hilgenfeld and Peiris, 2013; Paules et al., 2020). As with all previous outbreaks of new virus infections, we are being warned of impending doom concerning the new coronavirus, 2019-nCoV, which is now spreading globally.

## Characterization of Genes and Receptors of 2019-nCoV

At present, a total of 14 full-length genome sequences of 2019-nCoV have been submitted to GISAID and among them only one has been released on GenBank (Accession No: MN908947) (Ji et al., 2020). Coronaviruses are classified into four sub-families based on their genetic properties, alpha-, beta-, gamma-, and delta-coronavirus. SARS-CoV and MERS-CoV belong to the genus beta-coronavirus and can induce severe respiratory diseases in humans (Hilgenfeld and Peiris, 2013).

Phylogenetic analysis of genomic sequences of coronavirus deposited in the GenBank revealed that 2019-nCoV also belonged to the genus beta-coronavirus and displayed the closest linkage with two SARS-like coronaviruses from bat (Benvenuto et al., 2020; Guo et al., 2020; Wu et al., 2020; Zhou et al., 2020). Coronaviruses are enveloped viruses with a single-stranded RNA genome of positive polarity. The spike glycoprotein (S protein) is responsible for the receptor binding and membrane fusion. Angiotensin-converting enzyme 2 (ACE2) was identified as the receptor for SARS-CoV on the surface of human cells. Recent research demonstrated that ACE2 is also required for 2019-nCoV entry (Letko and Munster, 2020; Zhao et al., 2020b; Zhou et al., 2020). The binding affinity of receptor-binding domain (RBD) of S protein present in coronavirus is related to virus infectivity and pathogenicity. Another study showed that, in comparison to human SARS-CoV, the RBD of 2019-nCoV exhibited much lower affinity to ACE2 indicating a possible lower virulence of 2019-nCoV (Dong et al., 2020).

In addition to the major structural proteins, SARS-CoV genome encodes several accessory proteins and some of them play important roles with rapid evolution (Hilgenfeld and Peiris, 2013). *ORF8* encoded protein is an example of what can be learned. During the early stages of SARS-CoV outbreak, the *ORF8* gene coded for a single protein, but during the middle and late stages of the epidemic, the gene underwent a gradual 29 nt deletion and coded for two separate accessory proteins, ORF8a and ORF8b. It was hypothesized that this truncation event was responsible for the increased human-to human transmission efficiency that occurred during the late stages, triggering an epidemic. Interestingly, the 29 nt deletion in SARS-CoV led to an attenuating mutation with a reduction in virus replication levels (Muth et al., 2018; Oostra et al., 2007). Perhaps this attenuation was the potential reason why it was possible to eventually extinguish the outbreak. Unfortunately, the genome sequence of 2019-nCoV shows that *ORF8* is intact. If the gene does not undergo a gradual loss or mutation during subsequent virus circulation in humans, the outbreak could be more severe.

## Antiviral Therapy During 2019-nCoV Outbreak

In the early stages of an outbreak, most people do not have a reliable understanding of the susceptibility of the virus to antiviral drugs. Looking back on the medications used to fight SARS-CoV, initially ribavirin was recognized as a widely active antiviral drug that was effective against a range of RNA viruses but was of little use to SARS patients. The in vitro activities of ribavirin on the replication of SARS-CoV are highly variable, depending on the type of cells used for assays. Many SARS patients were treated with a combination of ribavirin and corticosteroids, given such poor prognosis linked to drug side effects (Cinatl et al., 2003a). In some countries, interferon alpha (IFN-α) is used in combination with immunoglobulin or thymosin and has a therapeutic effect (Zhao et al., 2003). In addition, there are reports that interferon beta (IFN-β) is significantly better than IFN-α and also that polyethylene glycol-modified IFN-α prevented SARS-CoV infection, reduced viral replication, and reduced histopathology during treatment (Cinatl et al., 2003b; Haagmans et al., 2004). To identify potential drugs for 2019-nCoV, the viral protease (Mpro) was modeled by Prof. Zihe Rao’s group at Tsinghua University, Beijing, China. They selected a series of potential drugs which exist in the market or the self-built database’s high-cost medicinal compounds and medicinal plant sources database compounds. Thirty drug candidates, consisting of biologically active natural products and traditional Chinese medicine drugs, that have the potential to show therapeutic effects against 2019-nCoV, were chosen for testing for the clinical treatment of pneumonia in patients infected with 2019-nCoV.

Based on previous anti-SARS studies and computer simulations, older drugs, like cinnamon thiamine and cyclosporin A (CsA), could be effective against 2019-nCoV. The immunosuppressive drug CsA prevents the nucleocapsid protein of the virus from binding to cyclophilin A (CypA) of the host cell, which has a peptidyl prolyl cis/trans isomerase (PPIase) activity, and a combination of interferon and CsA has been shown previously to significantly inhibit the replication and tissue damage caused by coronavirus infection in bronchi and lungs of humans. Pfefferle et al. detected specific interactions between CypA and SARS-nCoV non-structural protein 1 (Nsp1) by yeast-two-hybrid and other protein-protein interaction techniques. They also tested the drug CsA against a variety of coronaviruses and identified it to be a “pan-coronavirus inhibitor” (Haagmans et al., 2004). Since then, many inhibitors have been designed and synthesized to act against coronavirus, but few have experienced systematic toxicity in pre-clinical studies, and these compounds have not yet been used in clinical trials to prevent the recurrence of SARS-CoV or 2019-nCoV. This is one of the main difficulties in drug development.

## Early Warning Signs and Prediction of the 2019-nCoV Outbreak

Global public health concerns are focused against a number of known and well-characterized infections. However, the outbreak of pneumonia caused by a novel 2019-nCoV virus is a reminder that importance should be given to predicting the risk of novel virus infections in humans (Thompson, 2020). As this outbreak continues, more risk models are needed to help refine the risk assessment, which when pieced together with the emerging data will permit regular refinement and ensure optimal management of patients and healthy populations.

At present, complete genomes of different 2019-nCoV have been released, and the latest published information on these genomes provides peer-reviewed information that is urgently needed to improve the risk assessment and response, which occurs in real time (Chen et al., 2020b; Riou and Althaus, 2020). The possibility of human-to-human transmission of the virus is still under investigation. Understanding the transmission characteristics of 2019-nCoV and the potential for sustained human-to-human transmission is critically important for coordinating current screening and containment strategies, and for determining whether the outbreak constitutes a public health emergency of international concern. The situation is severe and it is urgent for us to need a better understanding towards this virus for further prevention and control. In order to better understand the early transmission pattern of 2019-nCoV, Julien et al. conducted a randomized simulation study of the early outbreak trajectories consistent with the epidemiological findings to date (Riou and Althaus, 2020). They suggested that a rapid establishment of sustained transmission chains from single cases cannot be ruled out, and at this stage, particularly attention should be given to the prevention of superspreading events. Chen et al. reported a mathematical model for simulating the transmission of the novel Wuhan Coronavirus, which is a Bats-Hosts-Reservoir-People transmission network model for simulating the potential transmission from the infection source to the humans (Chen et al., 2020a). In another study, a data-driven analysis in the early phase of the outbreak was conducted by Zhao et al. and a preliminary estimation of the basic reproduction number of 2019-nCoV was made. They estimated the transmissibility of 2019-nCoV via the basic reproduction number based on only the data from the early stages of the outbreak (Zhao et al., 2020a).

However, many other important aspects of virus biology, such as, whether the virus can travel across continents, the list of species it can infect and whether it can cause severe mortality, are much harder to forecast. Our premise is that the amount of sequencing data currently available and the latest advances in computing methods using that data will make it possible for the first time to generate virtual models of how viruses evolve in each environment and how those viruses behave. Such models have the potential to make risk assessments as they arise that can be used to inform policy and direct strategies to head off impending threats.

## Prospects for Future

To date, many problems remain to be solved, including the virus’ origin, extent, and duration of transmission in humans, its ability to infect animal hosts, its spectrum and pathogenesis of human infections, and an effective vaccine development.

At the heart of vaccine development is the question of immunology and it is crucial to understand the immunological questions associated with viral infections. The clinical characteristics and treatment of 2019-nCoV and SARS both suggested a serious problem of immunopathology, particularly in the lung mucosa, which is complex and unique. It might be due to the fact that a systematic protective immune response is not enough to protect against viral infection. Currently, one of the most dangerous but valuable experiments is to perform tests on immune cells in the blood and lungs of infected patients, preferably during different stages of viral infection. Data on clinical immunity can lay the foundation for future vaccine development. We need to be aware of the challenges and concerns that 2019-nCoV poses to our community. Every effort should be made to understand and control the disease.

## Footnotes

J.L. and W.L. conceived and prepared the manuscript and completed its revision. This work was supported by grants from the National Science and Technology Major Project (2018ZX10101004). J.L. is supported by the Youth Innovation Promotion Association of CAS (2019091).

The authors declare that they have no conflict of interest. This article does not contain any studies with human or animal subjects performed by any of the authors.
